# Factors influencing pathological complete response and tumor regression in neoadjuvant radiotherapy and chemotherapy for high-risk breast cancer

**DOI:** 10.1186/s13014-024-02450-5

**Published:** 2024-07-31

**Authors:** Jan Haussmann, Wilfried Budach, Carolin Nestle-Krämling, Sylvia Wollandt, Danny Jazmati, Bálint Tamaskovics, Stefanie Corradini, Edwin Bölke, Alexander Haussmann, Werner Audretsch, Christiane Matuschek

**Affiliations:** 1 Department of Radiation Oncology, Center for Integrated Oncology, Medical Faculty and University Hospital Düsseldorf , Heinrich Heine University, Aachen Bonn Cologne Düsseldorf (CIO ABCD), Dusseldorf, Germany; 2 Department of Senology, Sana-Kliniken Düsseldorf-Gerresheim, 40625 Dusseldorf, Germany; 3 Department of Radiation Oncology, University Hospital, LMU Munich, Munich, Germany; 4 Division of Physical Activity, Prevention and Cancer, German Cancer Research Center (DKFZ), 69120 Heidelberg, Germany; 5 Department of Senology and Breast Surgery, Breast Center at Marien Hospital Cancer Center, 40479 Dusseldorf, Germany; 6 Present Address: Department of Gynecological Oncological Rehabilitation, Asklepios Nordseesklinik, Sylt, Germany; 7 Department of Radiation Oncology, University Hospital OWL, Campus Bielefeld, Bielefeld, Germany

**Keywords:** Neoadjuvant radiotherapy, Neoadjuvant chemotherapy, pCR, Breast cancer, Breast response

## Abstract

**Background:**

Pathological complete response (pCR) is a well-established prognostic factor in breast cancer treated with neoadjuvant systemic therapy (naST). The determining factors of pCR are known to be intrinsic subtype, proliferation index, grading, clinical tumor and nodal stage as well as type of systemic therapy. The addition of neoadjuvant radiotherapy (naRT) to this paradigm might improve response, freedom from disease, toxicity and cosmetic outcome compared to adjuvant radiotherapy. The factors for pCR and primary tumor regression when neoadjuvant radiation therapy is added to chemotherapy have not been thoroughly described.

**Methods:**

We performed a retrospective analysis of 341 patients (cT1-cT4/cN0-N+) treated with naRT and naST between 1990 and 2003. Patients underwent naRT to the breast and mostly to the supra-/infraclavicular lymph nodes combined with an electron or brachytherapy boost. NaST was given either sequentially or simultaneously to naRT using different regimens. We used the univariate and multivariate regression analysis to estimate the effect of different subgroups and treatment modalities on pCR (ypT0/Tis and ypN0) as well as complete primary tumor response (ypT0/Tis; bpCR) in our cohort. Receiver operating characteristic (ROC) analysis was performed to evaluate the interval between radiotherapy (RT) and resection (Rx) as well as radiotherapy dose.

**Results:**

Out of 341 patients, pCR and pbCR were achieved in 31% and 39%, respectively. pCR rate was influenced by resection type, breast cancer subtype, primary tumor stage and interval from radiation to surgery in the multivariate analysis. Univariate analysis of bpCR showed age, resection type, breast cancer subtype, clinical tumor stage and grading as significant factors. Resection type, subtype and clinical tumor stage remained significant in multivariate analysis. Radiation dose to the tumor and interval from radiation to surgery were not significant factors for pCR. However, when treatment factors were added to the model, a longer interval from radiotherapy to resection was a significant predictor for pCR.

**Conclusions:**

The factors associated with pCR following naST and naRT are similar to known factors after naST alone. Longer interval to surgery might to be associated with higher pCR rates. Dose escalation beyond 60 Gy did not result in higher response rates.

## Introduction

Pathological complete response (pCR) is a well-established and pivotal prognostic factor in the management of breast cancer when treated with neoadjuvant systemic therapy (naST). Numerous factors that influence pCR have been identified, including intrinsic subtype, proliferation index, tumor grading, clinical tumor stage, clinical nodal status, and the type of systemic therapy [1–3]. The potential benefits of incorporating neoadjuvant radiotherapy (naRT) into this treatment paradigm are significant, encompassing improved treatment response, increased freedom from disease, reduced toxicity, and enhanced cosmetic outcomes [4, 5]. However, a comprehensive understanding of the influencing factors that determine pCR when radiation therapy is combined with naST remain unclear. Additionally, specific details of radiation therapy, such as the interval between radiotherapy and surgical resection, radiation dose to the tumor bed, and the extent of nodal target volume, may play integral roles in determining treatment outcomes.

This manuscript aims to provide a comprehensive analysis of all contributing factors affecting pCR in women who have undergone naST and naRT, with a particular emphasis on the details of radiation therapy. By focusing on these aspects, we aim to enhance our understanding of the multifaceted interplay between radiotherapy and chemotherapy in the preoperative setting for breast cancer, ultimately contributing to the optimization of treatment strategies and improved patient outcomes.

## Materials and methods

We searched the institutional database for patients receiving naRT and chemotherapy before their definitive breast cancer surgery between 1990 and 2003. All women that received axillary lymph node dissection (ALND) before the initiation of naRT and naST were excluded from the analysis. The long-term survival follow-up as well as quality of life and cosmetic results have already been published by our group [6–8].

Resection was performed as either a breast-conserving surgery with or without additional flap support or mastectomy with or without reconstruction. Axillary lymph node dissection was routinely performed. Tangential radiation therapy of the breast was applied using photon or cobalt therapy. Regional nodal irradiation to the axillary node level III and IV as well as the internal mammary node (IMN) was applied in selected patients. Axillary levels III and IV were treated with a separate supraclavicular field and IMNs were covered with an extension of the tangential breast fields. The dose was mainly 50 Gy to the breast with a 10 Gy boost to the tumor bed given as either electrons in 5 fractions or an interstitial HDR-brachytherapy boost of 10 Gy in one treatment. Brachytherapy was combined with one course of hyperthermia immediately before interstitial treatment. 2 Gy equivalent dose (EQD2) were calculated using an Alpha/Beta ratio of 3.7 [9].

Neoadjuvant chemotherapy (naCT) was given either sequentially (mostly before RT) or concurrently to RT with multiple regimens. The systemic therapy regimen was decided by the interdisciplinary team evaluating the patient and based on the historic standard protocols, individual risk factors as well the patients’ response to the ongoing therapy with clinical and ultrasound guided restaging. According to pathological outcome the interdisciplinary team also advised selected patients to undergo postneoadjuvant systemic therapy. For the analysis, chemotherapy schedules were categorized according to the current known efficacy into “standard” regimens (AC/EC + taxane, AC/EC + CMF, AC/EC + taxane + mitoxantrone) or “substandard” regimens (mitoxantrone only, AC/EC only, AC/EC + mitoxantrone, CMF +- mitoxantrone and other rarely used regimens). Patients with positive hormone receptor expression received endocrine therapy with tamoxifen, ovarian suppression, aromatase inhibitor or surgical ovariectomy. No Her2-targeted therapy was administered.

Based on the classification used in the early breast cancer trialists collaborative group (EBCTCG) meta-analysis, we also used the stratification of chemotherapy regimens into receipt of (1) no anthracycline or taxane, (2) anthracycline, no taxane, (3) anthracycline and taxane [10].

Biological breast cancer subtypes were defined according to hormone receptor status (estrogen or progesterone), HER2 positivity or lack of positivity for both receptors (triple negative). Retrospectively, the hormone receptor status was assessed by immunohistochemistry with cut-off values greater than 10 fmol/mg of protein regarded as positive [11]. HER2-positive breast cancer was subcategorized according in hormone receptor positive (HR+/HER2+) and hormone receptor negative subtype (HR-/HER2+). Hormone receptor positive and HER2 negative subtype was further categorized into luminal A-like and luminal B-like subtype according to grading, estrogen and progesterone receptor status as well Ki-67-value. Tumors with grade I and grade II with estrogen receptors (ER) and progesterone receptor (PR) expression above 20% and Ki-67 values below 14% were categorized as luminal A-like [11–15].

### Endpoint definition

We defined pCR as no residual tumor cells in the lymph nodes as well as the breast/chestwall with residual component strictly in situ according to Chevallier’s classification [16]. Breast pathological complete response (bpCR) was defined as primary tumor response with no invasive tumor left at the primary site (ypT0/ypTis).

### Statistical analysis

Patient characteristics are described using rates, means and medians for continuous and categorical variables. In order to assess the effect of various variables on pCR, we performed a cox regression analysis. For the multivariate analysis, we used all factors from the univariate cox regression analysis with *p* values < 0.1. Variables were entered simultaneously into the model. Age, radiation dose and time interval were entered as continuous variable into the analysis.

For the analysis of collinearity, we measured the variance inflation factor with a cut off of 10 and kept the clinically most relevant variable. In addition, we also tested the effect of adding clinically interesting and modifiable variables as radiation dose to the primary tumor, i.e., interval between radiotherapy and surgery, regional nodal irradiation, type of radiation boost, and neoadjuvant chemotherapy.

Further, we used ROC analysis to estimate the effect of the interval RT to Rx (time from first scheduled radiotherapy treatment day to date of primary tumor resection) and dose (in EQD2 (*a/b*=3.7Gy) for pCR and bpCR.

Two-sided *p*-values below the threshold of 0.05 were considered statistically significant. All statistical analyses were performed using SPSS (IBM Corp. Released 2016. IBM SPSS Statistics for Windows, Version 27.0. Armonk, NY: IBM Corp.) Figures and tables were created using Microsoft Excel for Microsoft Office 365 Pro Plus (Redmond, Washington, WA, USA).

The local ethics committee of the medical faculty of Düsseldorf University gave ethical approval of this retrospective study under the ID 4049.

## Results

The trial population consists of 341 patients in total. The baseline characteristics are described in Table 1. 106 women (31.1%) achieved a pCR and 133 women (39.0%) achieved a breast pCR after naRCTx.

**Table 1:** Overview of the baseline characteristics and treatment details

Characteristic	N (%)	Characteristic	N (%)	Characteristic	N (%)
Median age		Breast cancer subtype		Type of neoadjuvant chemotherapy	
< 45 y	66 (19.4)	HR + /HER2-Luminal A-like	52 (15.2)	None	15 (4.4)
45 y–55 y	129 (37.8)	HR + /HER2-Luminal B-like	162 (47.5)	Standard	98 (28.7)
> 55y	146 (42.8)	HR + /HER2 +	25(7.3)	Substandard	228 (66.9)
Resection type		HR + /HER2-	23 (6.7)	Type of Neoadjuvant Chemotherapy	
Breast conserving surgery	174 (51.0)	HR-/HER2-	55 (16.1)	None	15 (4.4)
Mastectomy	167 (49.0)	Unknown	24 (7.0)	EC/AC + CMF	82 /24.0)
Side primary tumor		Histology		EC/AC + Taxan	11 (3.2)
Right	154 (45.2)	Ductal	237 (69.5)	Standard Chemotherapy + 1 agent	5 (1.5)
Left	166 (48.7)	Mixed Ductal/Lobular	2 (0.6)	Mitoxantron	97 (28.4)
Unknown	21 (6.2)	Lobular	66 (19.4)	4–6 × EC	113 (33.1)
Clinical tumor stage		Other	11 (3.2)	AC/EC + Mitoxantron	2 (0.6)
T1	3 (0.9)	Unknown	25 (7.3)	CMF + Mitoxantron	6 (1.8)
T2	111 (32.6)	Median Interval RT to Rx	175 days	CMF	4 (1.2)
T3	149 (43.7)	Median Dose to Tumor Bed (EQD2(3.7))	60 Gy	Other	6 (1.8)
T4	78 (22.9)	Mean Dose to Tumor Bed (EQD2(3.7))	64 Gy	Type of neoadjuvant chemotherapy	
Clinical nodal stage		Type of breast radiotherapy		None	15 (4.4)
Negative	171 (50.1)	Cobalt Therapy	179 (52.5)	No Anthracycline or Taxane	109 (32.0)
Positive	170 (49.9)	Photon Therapy	156 (45.7)	Anthracycline, no Taxane	202 (59.2)
Stage		Regional nodal irradiation		Anthracycline and Taxane	15 (4.4)
I	1 (0.3)	None	46 (13.5)	Endocrine therapy	
IIA	66 (19.4)	Level 3 + 4	229 (67.2)	No endocrine therapy	76 (22.3)
IIB	121 (35.5)	Level 3 + 4 + IMN	40 (11.7)	Induction endocrine therapy	158 (46.3)
IIIA	74 (21.7)	IMN	9 (2.6)	Adjuvant endocrine therapy	85 (24.9)
IIIB	78 (22.9)	Unknown	17 (5.0)	Unknown	22 (6.5)
IIIC	1 (0.3)	Type of Boost RT			
Grading		Brachytherapy	99 (29.0)		
1	24 (7.0)	Cobalt	19 (5.6)		
2	138 (40.5)	Photon	35 (10.3)		
3	179 (52.5)	Electron	153 (44.9)		
Growth pattern		Mixed	12 (3.5)		
Unifocal	259 (76.0)	Unknown	22 (6.5)		
Multifocal	27 (7.9)	No Boost	1 (0.3)		
Multicentric	40 (11.7)				
Unknown	15 (4.4)				

Table 2 shows the rates of pCR and bpCR with the corresponding numbers and confidence intervals in different subgroups. pCR rates were numerically higher in younger women, more aggressive biological subtypes, smaller primary tumors, use of chemotherapy and longer interval from radiotherapy to resection. The analysis by subgroup did not show striking numerical differences by tumor side, clinical nodal status, growth patterns, tumor dose, regional nodal irradiation, type of chemotherapy or use of induction endocrine therapy.

**Table 2:** Rates of pathological complete response (ypT0/Tis ypN0) and breast pathological complete response (yT0/Tis) between different subgroups with the corresponding 95%-confidence intervals

Pathological complete response (ypT0/Tis ypN0)					
Subgroup	n Non-pCR	N pCR	Rate	CI—95% low	CI—95% high
All	235	106	0.31	0.26	0.36
*Age*					
Age < 45y	39	27	0.41	0.29	0.53
Age 45y − 55y	90	39	0.30	0.22	0.38
Age > 55y	106	40	0.27	0.20	0.35
*Resection type*					
Breast conserving surgery	106	68	0.39	0.32	0.46
Mastectomy	129	38	0.23	0.16	0.29
*Side breast cancer*					
Right	107	47	0.31	0.23	0.38
Left	115	51	0.31	0.24	0.38
Unknown	13	8	0.38	0.17	0.59
*Biological subtype*					
Luminal A	44	8	0.15	0.06	0.25
Luminal B	121	41	0.25	0.19	0.32
HR + HER2 +	18	7	0.28	0.10	0.46
HR − HER2 +	13	10	0.43	0.23	0.64
Triple negative	30	25	0.45	0.32	0.59
Unknown	9	15	0.63	0.43	0.82
*Histology*					
Ductal	158	79	0.33	0.27	0.39
Ductal/lobular	1	1	0.50	− 0.19	1.19
Lobular	49	17	0.26	0.15	0.36
Other	9	2	0.18	− 0.05	0.41
Unknown	18	7	0.28	0.10	0.46
*Clinical tumor stage*					
cT1	0	3	1.00	1.00	1.00
cT2	66	45	0.41	0.31	0.50
cT3	110	39	0.26	0.19	0.33
cT4	59	19	0.24	0.15	0.34
*Clinical nodal status*					
cN0	116	55	0.32	0.25	0.39
cN +	119	51	0.30	0.23	0.37
*Stage*					
I	0	1	1.00	1.00	1.00
IIA	41	25	0.38	0.26	0.50
IIB	78	43	0.36	0.27	0.44
IIIA	56	18	0.24	0.15	0.34
IIIB	59	19	0.24	0.15	0.34
IIIC	1	0	0.00	0.00	0.00
*Tumor grade*					
G1	20	4	0.17	0.02	0.32
G2	101	37	0.27	0.19	0.34
G3	114	65	0.36	0.29	0.43
*Growth pattern*					
Unifocal	178	81	0.31	0.26	0.37
Multifocal	17	10	0.37	0.19	0.55
Multicentric	30	10	0.25	0.12	0.38
Unknown	10	5	0.33	0.09	0.57
*Interval radiotherapy to resection*					
< Median time	121	45	0.27	0.20	0.34
> Median time	114	61	0.35	0.28	0.42
*Dose to tumorbed (EQD2 (3.7))*					
< 60 Gy (Median)	43	15	0.26	0.15	0.37
> 60 Gy (Median)	185	89	0.32	0.27	0.38
Unknown	7	4	0.36	0.08	0.65
*Dose to tumorbed (EQD2 (3.7))*					
< 64 Gy (Mean)	156	71	0.31	0.25	0.37
> 64 Gy (Mean)	72	33	0.31	0.23	0.40
Unknown	7	4	0.36	0.08	0.65
*Regional nodal irradiation*					
No RNI	27	19	0.41	0.27	0.56
Any RNI	208	87	0.29	0.24	0.35
No RNI	27	19	0.41	0.27	0.56
L3 − 4	164	65	0.28	0.23	0.34
L3 − 4 + IMN	29	11	0.28	0.14	0.41
IMN	4	5	0.56	0.23	0.88
Unknown	11	6	0.35	0.13	0.58
*Type of breast radiotherapy*					
Photon	125	54	0.30	0.23	0.37
Cobalt	107	49	0.31	0.24	0.39
Unknown	3	3	0.50	0.10	0.90
*Type of boost*					
Brachytherapy	69	30	0.30	0.21	0.39
Cobalt	17	2	0.11	− 0.03	0.24
Photon	23	12	0.34	0.19	0.50
Electron	102	51	0.33	0.26	0.41
Mixed	11	1	0.08	− 0.07	0.24
Unknown	13	9	0.41	0.20	0.61
No Boost	0	1	1.00	1.00	1.00
*Neoadjuvant chemotherapy*					
None	14	1	0.07	− 0.06	0.19
Standard	65	33	0.34	0.24	0.43
Substandard	156	72	0.32	0.26	0.38
*Neoadjuvant chemotherapy*					
None	14	1	0.07	− 0.06	0.19
EC/AC + CMF	50	32	0.39	0.28	0.50
4 × EC/AC + Taxan	10	1	0.09	− 0.08	0.26
3 drug combination	5	0	0.00	0.00	0.00
Mitoxantron	62	35	0.36	0.27	0.46
4 − 6 × EC	81	32	0.28	0.20	0.37
AC/EC + Mitoxantron	1	1	0.50	− 0.19	1.19
CMF + Mitoxantron	6	0	0.00	0.00	0.00
CMF	3	1	0.25	− 0.17	0.67
Other	3	3	0.50	0.10	0.90
*Neoadjuvant chemotherapy*					
None	14	1	0.07	− 0.06	0.19
No Anthracycline or Taxane	82	40	0.33	0.24	0.41
Anthracycline, no Taxane	140	64	0.31	0.25	0.38
Anthracycline and Taxane	12	3	0.20	0.00	0.40
*Induction endocrine therapy*					
No endocrine therapy	47	29	0.38	0.27	0.49
Induction endocrine therapy	112	46	0.29	0.22	0.36
Adjuvant endocrine therapy	63	22	0.26	0.17	0.35
Unknown	13	9	0.41	0.20	0.61

Breast pathological complete response (ypT0/Tis)					
Subgroup	N	N	Rate	CI—95% low	CI—95% high
	Non-bpCR	bpCR			
All	208	133	0.39	0.34	0.44
*Age*					
Age < 45y	30	36	0.55	0.43	0.67
Age 45 y–55 y	80	49	0.38	0.30	0.46
Age > 55 y	98	48	0.33	0.25	0.40
*Resection type*					
Breast conserving surgery	93	81	0.47	0.39	0.54
Mastectomy	115	52	0.31	0.24	0.38
*Side breast cancer*					
Right	90	64	0.42	0.34	0.49
Left	106	60	0.36	0.29	0.43
Unknown	12	9	0.43	0.22	0.64
*Biological subtype*					
Luminal A	44	8	0.15	0.06	0.25
Luminal B	110	52	0.32	0.25	0.39
HR + HER2 +	16	9	0.36	0.17	0.55
HR − HER2 +	8	15	0.65	0.46	0.85
Triple Negative	22	33	0.60	0.47	0.73
Unknown	8	16	0.67	0.48	0.86
*Histology*					
Ductal	138	99	0.42	0.35	0.48
Ductal/Lobular	1	1	0.50	− 0.19	1.19
Lobular	45	21	0.32	0.21	0.43
Other	8	3	0.27	0.01	0.54
Unknown	16	9	0.36	0.17	0.55
*Clinical tumor stage*					
cT1	0	3	1.00	1.00	1.00
cT2	58	53	0.48	0.38	0.57
cT3	95	54	0.36	0.29	0.44
cT4	55	23	0.29	0.19	0.40
*Clinical nodal status*					
cN0	105	66	0.39	0.31	0.46
cN +	103	67	0.39	0.32	0.47
*Stage*					
I	0	1	1.00	1.00	1.00
IIA	37	29	0.44	0.32	0.56
IIB	67	54	0.45	0.36	0.53
IIIA	48	26	0.35	0.24	0.46
IIIB	55	23	0.29	0.19	0.40
IIIC	1	0	0.00	0.00	0.00
*Tumor grade*					
G1	20	4	0.17	0.02	0.32
G2	94	44	0.32	0.24	0.40
G3	94	85	0.47	0.40	0.55
*Growth pattern*					
Unifocal	159	100	0.39	0.33	0.45
Multifocal	14	13	0.48	0.29	0.67
Multicentric	26	14	0.35	0.20	0.50
Unknown	9	6	0.40	0.15	0.65
*Interval radiotherapy to resection*					
< Median time	108	58	0.35	0.28	0.42
> Median time	100	75	0.43	0.36	0.50
*Dose to tumorbed (EQD2 (3.7))*					
< 60 Gy (Median)	37	21	0.36	0.24	0.49
> 60 Gy (Median)	165	109	0.40	0.34	0.46
Unknown	6	3	0.33	0.03	0.64
*Dose to tumorbed (EQD2 (3.7))*					
< 64 Gy (Mean)	143	84	0.37	0.31	0.43
> 64 Gy (Mean)	59	46	0.44	0.34	0.53
Unknown	6	3	0.33	0.03	0.64
*Regional nodal irradiation*					
No RNI	26	20	0.43	0.29	0.58
Any RNI	182	113	0.38	0.33	0.44
No RNI	26	20	0.43	0.29	0.58
L3 − 4	147	82	0.36	0.30	0.42
L3 − 4 + IMN	24	16	0.40	0.25	0.55
IMN	2	7	0.78	0.51	1.05
Unknown	9	8	0.47	0.23	0.71
*Type of breast radiotherapy*					
Photon	114	65	0.36	0.29	0.43
Cobalt	92	64	0.41	0.33	0.49
Unknown	2	4	0.67	0.29	1.04
*Type of boost*					
Brachytherapy	57	42	0.42	0.33	0.52
Cobalt	16	3	0.16	− 0.01	0.32
Photon	20	15	0.43	0.26	0.59
Electron	92	61	0.40	0.32	0.48
Mixed	11	1	0.08	− 0.07	0.24
Unknown	12	10	0.45	0.25	0.66
No Boost	0	1	1.00	1.00	1.00
*Neoadjuvant chemotherapy*					
None	13	2	0.13	− 0.04	0.31
Standard	54	44	0.45	0.35	0.55
Substandard	141	87	0.38	0.32	0.44
*Neoadjuvant chemotherapy*					
None	13	2	0.13	− 0.04	0.31
EC/AC + CMF	39	43	0.52	0.42	0.63
4 × EC/AC + Taxan	10	1	0.09	− 0.08	0.26
3 drug combination	5		0.00	0.00	0.00
Mitoxantron	54	43	0.44	0.34	0.54
4 − 6 × EC	77	36	0.32	0.23	0.40
AC/EC + Mitoxantron	1	1	0.50	− 0.19	1.19
CMF + Mitoxantron	5	1	0.17	− 0.13	0.46
CMF	1	3	0.75	0.33	1.17
Other	3	3	0.50	0.10	0.90
*Neoadjuvant chemotherapy*					
None	13	2	0.13	− 0.04	0.31
No Anthracycline or Taxane	60	49	0.45	0.36	0.54
Anthracycline, no Taxane	123	79	0.39	0.32	0.46
Anthracycline and Taxane	12	3	0.20	0.00	0.40
*Induction endocrine therapy*					
No endocrine therapy	37	39	0.51	0.40	0.63
Induction endocrine therapy	103	55	0.35	0.27	0.42
Adjuvant endocrine therapy	56	29	0.34	0.24	0.44
Unknown	12	10	0.45	0.25	0.66

**Fig. 1:**
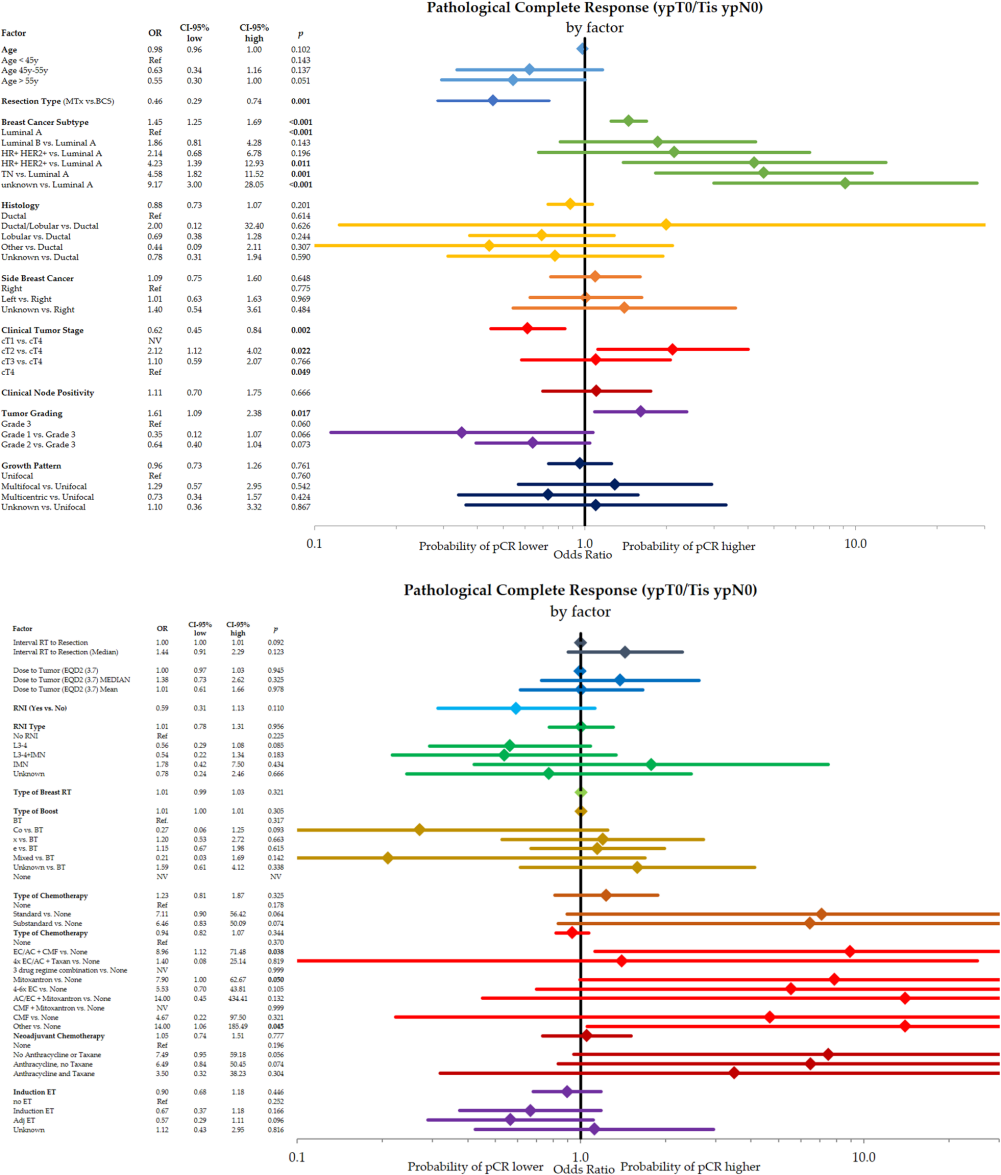
Univariate analysis of different factors for pathological complete response (ypT0/Tis ypN0). Shown are different factors and subgroups with the odds ratios for the probability of a pathological complete response with the corresponding 95%-intervals. Higher odds ratios indicate a higher probability of achieving a pCR

Figure 1 analyzes the effect of different subgroups on pCR using a univariate assessment. Resection type, breast cancer subtype, primary tumor T-stage, and grading were significantly associated with the result of reaching a pCR. Histological subtype, side of breast cancer, growth pattern, interval from RT to resection, dose to tumor, type of radiation boost as well as type and classification of systemic therapy were not significantly associated with pCR.

**Fig. 2:**
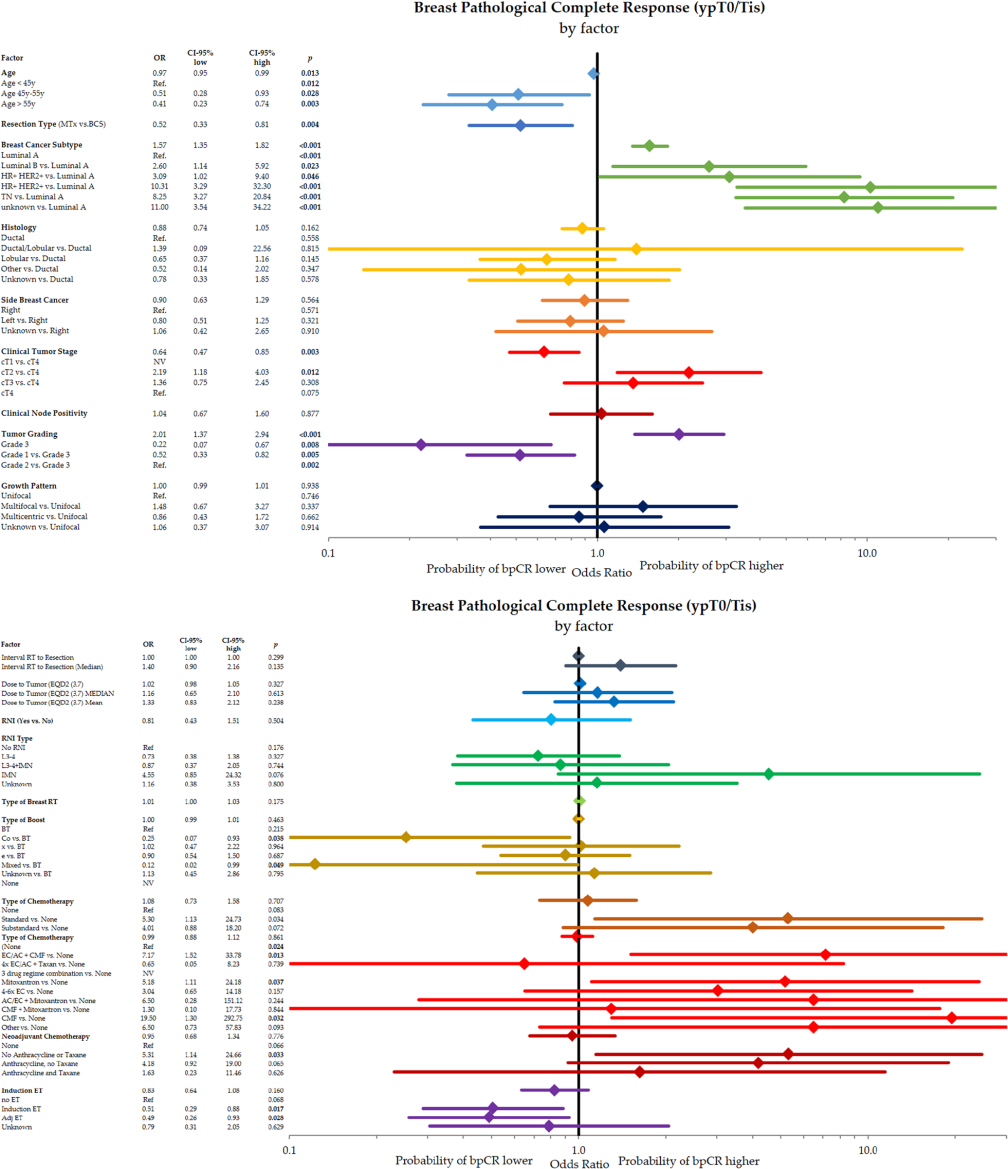
Univariate analysis of different factors for breast pathological complete response (ypT0/Tis). Shown are different factors and subgroups with the odds ratios for the probability of a pathological complete response with the corresponding 95%-intervals. Higher odds ratios indicate a higher probability of achieving a bpCR

Further, we analyzed factors that were associated with the pCR only in the breast (ypT0/Tis) in different subgroups (Fig. 2). In the univariate analysis, we detected that significant factors for bpCR were age, resection type, breast cancer subtype, clinical tumor stage, and grading.

Table 3 further shows the results of the multivariate analysis using different models. The diagnosis of a pCR was independently associated with resection type (*p* = 0.032), lower clinical tumor stage (*p* < 0.001) and breast cancer subtype (*p* = 0.009). The endpoint of bpCR was associated with subtype (*p* < 0.001), clinical tumor stage (*p* = 0.007) and grading (*p* = 0.027). When adding dose and interval to the models, subtype, stage and interval remained predictive for pCR. For bpBR, subtype, stage and grading remained predictive factors in the multivariate analysis. Dose to the primary tumor had no significant effect. In model 3 we added dose, interval, type of regional nodal irradiation, boost type and type of chemotherapy to the model. The multivariate analysis of pCR again showed subtype, stage and interval, as predictive variable whereas for bpCR subtype, stage and grading remained the predictive factor. All other factors included exerted no significant influence in the multivariate analysis.

**Table 3:** Multivariate analysis of pathological complete response and breast pathological complete response using three different models with odds ratios and confidence intervals

Pathological complete response (ypT0/ypTis ypN0)					Breast pathological complete response (yT0/Tis)				
Model 1	OR	CI—95% low	CI—95% high	*p*	Model 1	OR	CI—95% low	CI—95% high	*P*
Age	–				Age	0.98	0.96	1.01	0.182
Resection type (MTx vs. BCS)	0.57	0.34	0.95	**0.032**	Resection type (MTx vs. BCS)	0.62	0.38	1.03	0.066
Breast cancer subtype	1.41	1.20	1.65	** < 0.001**	Breast cancer subtype	1.48	1.26	1.74	** < 0.001**
Clinical tumor stage	0.62	0.44	0.89	**0.009**	Clinical Tumor Stage	0.63	0.44	0.88	**0.008**
Grading	1.42	0.92	2.21	0.117	Grading	1.73	1.13	2.66	**0.012**
constant	0.60			0.456	Constant	0.96			0.963

Model 2 (with Dose and Interval)	OR	CI—95% low	CI—95% high	*p*	Model 2 (with Dose and Interval)	OR	CI-95% low	CI-95% high	*p*
Age	–				Age	0.98	0.96	1.01	0.209
Resection type (MTx vs. BCS)	0.62	0.36	1.07	0.085	Resection type (MTx vs. BCS)	0.68	0.40	1.15	0.146
Breast cancer subtype	1.45	1.23	1.72	** < 0.001**	Breast cancer subtype	1.52	1.28	1.79	** < 0.001**
Clinical tumor stage	0.60	0.41	0.87	**0.008**	Clinical tumor stage	0.60	0.41	0.87	**0.007**
Grading	1.52	0.93	2.49	0.094	Grading	1.72	1.07	2.78	**0.027**
Interval RT to Rx	1.00	1.00	1.01	**0.043**	Interval RT to resection	1.00	1.00	1.01	0.194
Dose to tumor (EQD2 (3.7))	0.99	0.95	1.03	0.495	Dose to tumor (EQD2 (3.7))	1.00	0.96	1.04	0.923
Constant	0.61			0.709	Constant	0.68			0.790

Model 3 (with dose, interval, RNI type, Boost, CTx)	OR	CI—95% low	CI—95% high	*p*	Model 3 (with dose, interval, RNI type, boost, CTx)	OR	CI—95% low	CI—95% high	*p*
Age	–				Age	0.98	0.96	1.01	0.243
Resection type (MTx vs. BCS)	0.62	0.36	1.07	0.087	Resection type (MTx vs. BCS)	0.68	0.40	1.15	0.148
Breast cancer subtype	1.46	1.23	1.73	** < 0.001**	Breast cancer subtype	1.53	1.29	1.81	** < 0.001**
Clinical tumor stage	0.60	0.41	0.87	**0.007**	Clinical tumor stage	0.59	0.41	0.86	**0.006**
Higher grading	1.55	0.93	2.58	0.097	Higher grading	1.73	1.05	2.86	**0.032**
Interval RT to Rx	1.00	1.00	1.01	**0.042**	Interval RT to resection	1.00	1.00	1.01	0.197
Dose to tumor (EQD2 (3.7))	0.99	0.94	1.03	0.527	Dose to tumor (EQD2 (3.7))	1.00	0.95	1.04	0.911
RNI type	1.01	0.99	1.02	0.370	RNI type	1.01	0.99	1.02	0.321
Type of boost	1.01	0.99	1.02	0.430	Type of boost	1.00	0.99	1.02	0.682
Type of neoadjuvant chemotherapy	1.00	0.59	1.68	0.984	Type of neoadjuvant chemotherapy	1.03	0.62	1.72	0.908
Constant	0.58			0.772	Constant	0.61			0.804

Significant values are highlighted in bold

Model 1 used significant factors from the univariate analysis whereas models 2 and 3 added potentially modifiable factors in the clinical decision process. MTx, Mastectomy; BCS, Breast conserving surgery, EQD2 (3.7), 2Gy-equivalent dose with an alpha/beta of 3.7; RNI, Regional nodal irradiation; RT, Radiotherapy; Rx, Resection

The linear analysis of pCR and bpCR by interval and RT dose is shown in Table 4. Higher biological radiation doses were not significantly associated with pCR and bpCR response (*p*=0.908 and *p*=0.433). Increasing time interval from radiotherapy to resection was partly associated with pCR rate (*p*=0.070) but not with bpCR (*p*=0.179).

**Table 4:** ROC Analysis of pCR and bpCR by 2 Gy equivalent dose using an alpha/beta of 3.7 and time interval from radiotherapy to surgical resection

Analysis	ROC	CI—95% low	CI—95% high	*p*
pCR by RT Dose (EQD2 (3.7))	0.50	0.44	0.57	0.908
Breast pCR by RT Dose (EQD2 (3.7))	0.53	0.46	0.59	0.433
pCR by RT-OP Interval	0.56	0.50	0.63	0.070
Breast pCR by RT-OP Interval	0.54	0.48	0.61	0.179

Additional analyses of pCR and bpCR by year of treatment time, categorical dose and time interval from RT to resection are shown in the appendix tables 6, 7, 8.

## Discussion

In this study, we present one of the largest series of neoadjuvant radiotherapy combined with chemotherapy for high-risk breast cancer. Our analysis revealed noteworthy pathological complete response (pCR) rates in the breast and lymph nodes (31%) and breast alone (bpCR) (39%) when using older radiotherapy and chemotherapy regimens.

The regression of the primary tumor by neoadjuvant systemic therapy is a crucial prognostic factor in breast cancer. Pathological complete response in both the primary breast tumor and draining lymph nodes holds the best prognostic value for high-risk breast cancer, surpassing the prognostic value of the response in the breast tumor alone [1, 8].

To evaluate the contributing factors for response after combined systemic therapy and radiotherapy, we conducted a comprehensive analysis across different subgroups, taking into consideration well-established factors such as age, clinical tumor stage, breast cancer subtype, grading, and the type and intensity of chemotherapy [13].

Similar to neoadjuvant systemic therapy alone, our analysis identified intrinsic subtype and clinical tumor stage as independent significant factors for pCR. Additionally, tumor grading was a significant factor in bpCR. The lack of a significant influence of clinical nodal status and type of systemic therapy on pCR or bpCR is most likely explained by the retrospective nature of the analysis, which can suffer from selection bias. This can be illustrated in the pCR rates by chemotherapy type where the most intense chemotherapy regimens had lower pCR numbers.

The addition of radiotherapy in the neoadjuvant concept was directed at the whole breast with or without the level 3 und 4 axillary lymph nodes and the internal mammary nodes. The dose to the axillary lymph nodes level I and II was mainly incidental and not standardized. Thus, the difference in factors adding to pathological complete responses to the breast compared to breast + lymph nodes might inform us about the added value of RT compared to the effect of chemotherapy alone. Here, only tumor grading differed between the two endpoints.

We did not observe any numerical differences in clinically node positive and negative patients and our multivariate analysis of prognostic factors showed that pCR and bpCR are influenced by similar factors irrespective of the preoperative addition of radiotherapy. These findings supports the interpretation of pCR as a biologic characteristic rather than therapy dependent factor.

Remarkably, our study is the first to demonstrate that naRCT for breast cancer yields response parameters similar to naST without RT. Moreover, RT-associated factors, including dose, target volume, and type of boost, did not exert a significant impact on response rates.

One notable finding is the possible correlation we observed between the treatment interval between radiotherapy and surgical resection and the likelihood of achieving a pCR. This observation aligns with similar trends in other oncological entities, such as esophageal and rectal cancer, where the timing of radiotherapy in the preoperative treatment paradigm has been studied extensively. For example, in rectal cancer, a 10-week interval between radiotherapy and surgery appears optimal for pCR rates, while longer intervals do not adversely affect clinical outcomes but do not tend results in higher response rates [17, 18]. In esophageal cancer, the effect of timing is less consistent, with some studies suggesting a small, non-significant increase in histological complete response with a longer interval [19]. However, results vary in the literature, and in other studies, longer intervals did not result in higher pCR rates [15].

When neoadjuvant chemotherapy is administered as a preoperative therapy in breast cancer, increasing the interval between chemotherapy and surgery has not been shown to influence the pCR rates [20–22]

Contrary to some malignancies like esophagus and rectal cancer, where some studies describe a relationship between radiation dose and response, our study did not reveal any linear influence of dose on pCR response [23–31]. Dose escalation beyond 60 Gy, achieved mainly through combined brachytherapy and hyperthermia, did not significantly impact pCR rates. Notably, most patients in our study received radiation doses well above 50 Gy, with only a small percentage treated below 56 Gy. The addition of chemotherapy might also have mitigated any additional dose-related effects.

This observation is supported by older studies investigating the influence of different radiation doses in resectable and unresectable breast cancer. These trials reported that breast doses above 60 Gy but no beyond 80 Gy were required to achieve acceptable local control rates [32–36]. Our results are in line with the IMPORT-HIGH trial where a simultaneously integrated boost was tested in higher risk breast cancer in the adjuvant setting after BCS [37]. The authors did not observe a significant effect for local control beyond a dose of 48 Gy in 15 fractions which corresponds to an EQD2(3.7) of 58.1 Gy.

Other publications have further described additional factors that are contributing to the aim of achieving a pCR. Beyond the known and well-investigated demographic factors like age and tumor-specific biological factors there are certainly other variable that can influence pCR rates.

In our study, the median interval between radiotherapy and surgery was 175 days, possibly contributing to the observed favorable pCR rates. Furthermore, tumor-specific attributes like hormone receptor expression and proliferation indexes [38, 39][22] are import factors to achieve a pCR. Other factors involve tumor gene expression profiles like microRNA patterns [40], NRF2 [41], PIK3CA [42] and distinct gene expression classifiers [43–47]. The surrounding tumor stroma ratio [48, 49] might also play a crucial role for the activity of tumor-infiltrating lymphocytes (TILs) [50–55]. The pattern of residual disease (concentric, scattered) might also influence this endpoint [49]. Another component is the host immune system measured by inflammatory markers in the tissue [56–59] as well as in the blood serum [38, 39].

To contextualize our results in the context of cohorts treated with neoadjuvant systemic therapy alone, we compared our pCR and bpCR rates to large meta-analyses, as shown in Table 5. The CTNeoBC meta-analysis in patients treated with naST between 1990 and 2011 found a strong prognostic impact of pCR in different subgroups. The EBCTCG meta-analysis assessed the clinical complete response (no evidence of disease after naST) which we compared to the bpCR rates. Overall, there seems to be a numerical improvement of response rates (pCR or bpCR) of around 10% with the addition of naRT.

**Table 5:** Comparison of pCR and bpCR in different subgroups of this analysis to large meta-analysis databases

Subgroup	CTNeoBC (1)	This trial	Subgroup	EBCTCG (10)	This trial
	pCR	pCR		CCR	bpCR (yT0/Tis)
cT1	18.3% (15.7–21.2%)	100.0%	cT1	34.6%	100.0%
cT2	19.9% (19.0–20.9%)	40.5% (31.4–49.7%)	cT2	29.7%	47.7% (38.5–57.0%)
cT3	13.0% (11.7–14.3%)	26.2% (19.1–33.2%)	cT3-4	13.3%	29.5–36.2%
cT4	14.5–16.0% (12.1–19.6%)	24.4% (14.8–33.9%)			(19.4–44.0%)
cN0	18.8% (17.9–19.8%)	32.2% (25.2–39.2%)	cN0	28.6%	38.6% (31.3–45.9%)
cN +	16.9% (15.9–17.9%)	30.0% (23.1–36.9%)	cN +	27.2%	39.4% (32.1–46.8%)
Ductal	15.5% (14.7–16.3%)	33.3% (27.3–39.3%)			
Lobular	7.8% (6.4–9-4%)	25.8% (15.2–36.3%)			
Grade I	7.8% (6.4–9-4%)	16.7% (1.8–31.6%)	Grade I	20.9%	16.7% (1.8–31.6%)
Grade II	12.3% (11.3–13-3%)	26.8% (19.4–34.2%)	Grade II	36.0%	31.9% (24.1–39.7%)
Grade III	25.8% (24.3–27.4%)	36.3% (29.3–43.4%)	Grade III	44.6%	47.5% (40.2–54.8%)
HR + /HER2-G1/2	7.5% (6.3–8.7%)	15.4% (5.6–25.2%)	ER + G1-2	31.4%	15.4% (5.6–25.2%)
HR + /HER2-G3	16.2% (13.4–19-3%)	25.3% (18.6–32.0%)	ER + G3	34.9%	32.1% (24.9–39.3%)
HR + /HER2 + (no H)	18.3% (15.5–21.3%)	28.0% (10.4–45.6%)	ER- G1-2	37.2%	NA
HR-/HER2 + (no H)	30.2% (26.0–34.5%)	43.5% (23.2–63.7%)	ER- G3	52.9%	NA
Triple negative	33.6% (30.9–36.4%)	45.5% (32.3–58.6%)			
			Age < 45 y	29.8%	54.5% (42.5–66.6%)
			Age 45–55 y	29.0%	38.0% (29.6–46.4%)
			Age > 55 y	25.8%	32.9% (25.3–40.5%)
			No Anthracycline or Taxane	18.5%	45.0% (35.6–54.3%)
			Anthracycline, no Taxane	26.0%	39.1% (32.4–45-8%)
			Anthracycline and Taxane	41.0%	20.0% (− 0.2–40.2%)

95% confidence interval shown in brackets. *CCR* Clinical complete response, *H* Trastuzumab

Interestingly this number is also present in the trial treating adenocarinoma of the gastro-esophageal junction to chemotherapy alone or radiochemotherapy where naRT improved pCR rates from 2 to 16% [60, 61].

It is important to acknowledge the limitations of our analysis, primarily from its retrospective design, which carries the risk of selection bias. Additionally, our cohort is unique in terms of the cytotoxic agents used, the frequent use of combined hyperthermia and brachytherapy radiation boosts, and the relatively long median time interval of 175 days between RT and resection.

As for the observed lack of a dose-response relationship, this may be attributed to the already high tumor doses delivered in our trial, with median and mean doses of 60 and 64 Gy. It is worth noting that current pCR rates are higher with the addition of immune checkpoint inhibition, HER2-targeted therapies, and the combination of multiple cytotoxic agents.

In order to further elucidate the role of radiation therapy in the multidisciplinary treatment of high-risk breast cancer, the next step should be a randomized controlled trial comparing neoadjuvant radiotherapy to adjuvant radiotherapy. This trial should explore whether the observed improved response rates translate into longer disease-free and overall survival outcomes. Fortunately, this trial is about to open at multiple sites across Germany (NCT04261244). Additionally, other trials are investigating whether radiotherapy before mastectomy, with simultaneous breast reconstruction using implants or autologous flap reconstruction, can improve breast reconstruction outcomes and reduce reconstruction-related adverse events.

## Conclusion

In conclusion, our study contributes valuable insights into the combination of neoadjuvant radiotherapy and chemotherapy for high-risk breast cancer. While the addition of radiotherapy did not significantly alter the factors contributing to pCR, the timing of radiotherapy in the preoperative setting emerged as a modestly correlated factor. The lack of a linear dose-response relationship and the already high tumor doses delivered suggest that further dose escalation may not be beneficial.

## Data Availability

The datasets generated and/or analysed during the current study are not publicly available due to confidentiality and privacy concerns but numerical data are available from the corresponding author on reasonable request.

## Appendix

See Tables 6, 7, 8.

**Table 6:** Analysis of pCR and bpCR by treatment time

Treatment time/endpoint	All		1991–1993		1994–1996		1997–1999		2000–2003		*p*
pCR	n	n pCR	n	n pCR	n	n pCR	n	n pCR	n	n pCR	
	341	31.1%	90	36.7%	121	28.1%	118	28.0%	12	50%	0.232
bpCR	n	n bpCR	n	n bpCR	n	n bpCR	n	n bpCR	n	n bpCR	
	341	39.0%	90	31.6%	121	37.2%	118	33.9%	12	50%	0.234

**Table 7:** Analysis of pCR and bpCR by categorized dose

Dose/ Endpoint	All		< 50 Gy		50–55 Gy		55–60 Gy		60–65 Gy		65–70 Gy		> 70 Gy		*p*
pCR	n total	n pCR	n	n pCR	n	N pCR	n	n pCR	n	n pCR	n	n pCR	n	n pCR	
	330	30.9%	1	100%	2	50.0%	186	31.2%	38	28.9%	5	20.0%	98	30.6%	0.766
bpCR	n total	n pCR	n	n pCR	n	n pCR	n	n pCR	n	n pCR	n	n pCR	n	n pCR	
	330	38.8%	1	100%	2	50.0%	186	37.1%	38	34.2%	5	40.0%	98	42.9%	0.773

**Table 8:** Analysis of pCR and bpCR by time interval between radiotherapy and resection

Endpoint/ time interval	pCR	n pCR	n total	bpCR	n bpCR	n total
8–12 weeks	14.3%	1	7	28.6%	2	7
12–16 weeks	31.4%	11	35	42.9%	15	35
16–20 weeks	21.4%	12	56	26.8%	15	56
20–24 weeks	30.9%	17	55	36.4%	20	55
24–28 weeks	30.6%	15	49	42.9%	21	49
32–36 weeks	25.0%	9	36	38.9%	14	36
40–44 weeks	42.9%	12	28	53.6%	15	28
44–48 weeks	44.1%	15	34	44.1%	15	34
48–52 weeks	33.3%	4	12	41.7%	5	12
52–56 weeks	16.7%	1	6	33.3%	2	6
56–60 weeks	75.0%	3	4	75.0%	3	4
60–64 weeks	33.3%	3	9	33.3%	3	9
64–68 weeks	0.0%	0	1	0.0%	0	1
68–72 weeks	50.0%	2	4	50.0%	2	4
72–76 weeks	0.0%	0	3	0.0%	0	3
88–92 weeks	100.0%	1	1	100.0%	1	1

## Abbreviations

AC/EC: Doxorubicin + cyclophosphamide/epirubicin + cyclophosphamide
ALND: Axillary lymph node dissection
BCS: Breast conserving surgery
bpCR: Breast pathological complete response
CMF: Cyclophosphamide + methotrexate + 5-fluorouracil
EBCTCG: Early breast cancer trialists collaborative group
EQD2: 2 Gy equivalent dose
ER: Estrogen receptors
Gy: Gray
Her2: Human epidermal growth factor receptor 2
H: Trastuzumab
HR: Hormone receptor
IMN: Internal mammary nodes
MTx: Mastectomy
naRCT: Neoadjuvant radiochemotherapy
naRT: Neoadjuvant radiotherapy
naST: Neoadjuvant systemic therapy
OR: Odds ratio
pCR: Pathological complete response
PR: Progesterone receptor
ROC: Receiver operating characteristic
RT: Radiotherapy
Rx: Resection
